# The Effects of Herbs and Fruits on Leukaemia

**DOI:** 10.1155/2014/494136

**Published:** 2014-08-27

**Authors:** Tayebeh Azam Saedi, Sabariah Md Noor, Patimah Ismail, Fauziah Othman

**Affiliations:** ^1^Institute of Bioscience, Universiti Putra Malaysia, Malaysia; ^2^Department of Human Anatomy, Faculty of Medicine and Health Sciences, Universiti Putra Malaysia (UPM), 43400 Serdang, Selangor Darul Ehsan, Malaysia; ^3^Department of Pathology, Faculty of Medicine and Health Sciences, Universiti Putra Malaysia (UPM), 43400 Serdang, Selangor Darul Ehsan, Malaysia; ^4^Department of Biomedical Sciences, Faculty of Medicine and Health Sciences, Universiti Putra Malaysia (UPM), 43400 Serdang, Selangor Darul Ehsan, Malaysia

## Abstract

In developing countries, herbal therapy is the first and basis form of treatment for most types of diseases. About 75–80% of the world's population prefers herbal therapy as a major treatment due to its better adequacy and satisfactoriness, which enhance human body's symmetry with minimal side effects. Fruits and plants have been presented from the past as promising tools in becoming a natural anticancer agents. Many of these plant extracts are currently used in cancer therapy and prevention. This review paper will particularly explore and emphasize on herbs and fruits used in the treatment of the leukaemia.

## 1. Introduction

Leukaemia has been recognized since 1845, when a report was published on a patient who died of the disease which have shown an amplified number of blood cells. Twenty years later it was found that diagnosis of leukaemia could be possible through bone marrow puncture [1]. Leukaemia is one of the most common types of cancer worldwide. Decreased in incidence of infectious diseases and increased human life span caused prevalence of leukaemia. The use of therapeutic herbs in developing countries as cures against leukaemia is prominent [2].

Currently, Denmark has the highest incidence of leukaemia (29% incidence per 100,000 individuals) in the world [3]. It was reported that 21, 464 cancer cases (9,400 males and 12,064 females) were diagnosed among Malaysians of all ages in 2003 and the incidence rate for cancer in Malaysia in the year 2003 was 134.3 per 100, 1000 males and 154.2 per 100,000 females [4].

Among of 21, 464 cancer cases the incidence rates of lymphoid leukaemia in Malaysia for both males and females were 2.8 and 1.7 per 100.000 populations, respectively. On the other hand, the incidence rates of myeloid leukaemia in Malaysia for males and females were 3.0 and 2.7 per 100.000 populations, respectively [5].

Leukaemia is diagnosed 10 times more often in adults than in children and more common in males than females [6]. In the year 2000, nearly 2, 56,000 children and adults around the world developed some form of leukaemia, and 2,09,000 died from it [7]. Many factors are related to the development of leukaemia: prior chemotherapy, hereditary syndromes (Down syndrome), ionizing radiation, viruses infection, and smoking. People with leukaemia are treated with a combination of treatment including chemotherapy (main treatment), antibiotic, blood transfusion, radiation therapy, and bone marrow transplantation. Although these treatments have prolonged the survival rate of patients with leukaemia. Some of these treatments are difficult to handle [8]. Thus, there is a need to seek for other remedies in combating this disease. Therefore, this review paper is aimed at giving an overview of herbs and fruits which have been demonstrated as therapeutic agent for leukaemia.

## 2. Types of Leukaemia

According to pathological feature, there are four major types of leukaemia. The acute leukaemia is divided into acute myeloid leukaemia (AML) and acute lymphoblastic leukaemia (ALL). Acute promyelocytic leukaemia (APL) is a subtype of acute myeloid leukaemia. The chronic leukaemias are divided into chronic myeloid leukaemia (CML) and chronic lymphocytic leukaemia (CLL) [9].

## 3. Privilege of Herbal Therapy

Medicinal herbs have symbolized safety in contrast to the synthetic treatment (chemotherapy and radiotherapy) [10]. They play an essential role in the treatment of cancer [11–14]. In patients with cancer the usage of complementary and alternative medicine is the first desirable treatment with slight side effects and lesser adverse effects as compared to the synthetic ones (Kinghorn et al., 2003).

## 4. The History of Traditional Medicine

Based on Chinese conception, the origin of cancer is considered to be mainly due to lack of “Zheng Qi,” immune system deficiency and accumulation of “Xie Qi,” pathogenic factors. Experimental and clinical Chinese medicine therapies have demonstrated that many herbal formulations are effective for treating cancer in various stages [15]. A plant used in treating diseases is as old as civilization [16]. Traditional medicines are still a major part of habitual treatment of different diseases [17]. Plants are considered as one of the main sources of biologically active materials. Based on epidemiological studies, some foods, such as dietary fiber, vegetables, fruit and soy, have been acting as chemopreventive agents on the gastrointestinal tract [18]. The accumulated evidence showed an association between consumption of fruit and vegetables with risk of cancer [19]. Recent records reported that medicinal herbs are used by 80% of people living in rural areas as principal health care system [20]. In the Middle East region, 700 species of the identified plants are well-known for their medicinal values [21]. Compared with chemical synthesis, plant derived natural products represent an attractive source of biologically active agents since they are naturally and available at affordable prices [22]. For years, scientists were examining to cure cancer by chemically synthesized or natural one. In the past, the researcher has been concentrated on the use of crude extracts or a combination of different phytochemicals to treat the cancer. This approach is based on finding that the synergistic effect of the different plant metabolites in the crude extract, second, is the multiple points of intervention of such extracts which leads to controlling of different diseases [23].

For many years, medicinal plants have been used to treat different diseases [24]. Records of fossil documented the use of medicinal plants by humans before 60,000 years [16]. The Mediterranean has been characterized by high inventory of medicinal herbs used by local traditional therapists to treat different diseases [21]. During the Ottoman Empire and following the Byzantine traditions, hospitals consumed medicinal plants and remedies originating from classical Greek and folk medicinal practice to cure patients [25]. Herbalist studies in Palestine have shown that liver, digestive tract, respiratory system, skin, cancer, and other diseases can be treated by nearly 129 plant species (Azaizeh et al., 2003). On the other hand, the high diversity of plant species in Jordan is an opportunity for scientists to discover the distribution of therapeutic plants.

Seventy-nine plant species are still used in traditional medicine in the Showbak region (South of Jordan) while forty-six are part of the popular medicine in the Ajloun Heights region (North of Jordan). Some of these plants are used in both regions [26, 27]. Recently, reports showed that in the Arab traditional medicine, less than 200–250 plant species are for treating different diseases [28], as compared to more than 700 species that were used in previous decades [21]. The high rate of plant extinction on the earth necessitates an increase in the efforts to study plant natural products for their potential to provide treatment for different afflictions. Plants and their extracts are therapeutically superior to their single isolated constituents. They are generally plentiful, low cost, and relatively nontoxic in clinical practice. So, their medicinal properties are under extensive investigation, as their use has become a major part of complementary and alternative medicines (CAMs) [29]. Based on literature, there are types of herbs, fruits and their compounds acted as an inhibitor of carcinogen formation, blockers of carcinogen interaction, and suppressor of tumor progression as in the Tables 1 and 2.

### 4.1. *Hibiscus cannabinus* (Kenaf)

It contains bioactive components such as tannins, saponins, polyphenolics, alkaloids, fatty acids, phospholipids, tocopherol, and phytosterols [44]. Seed oil of kenaf is a unique and rich source of bioactive compounds with high antioxidative and anticancer properties [45, 46]. In addition, it has been reported that this herb inhibits the action of carcinogenic chemicals in colon cancer induction in rats [47] and also kills ovarian cancer cells through apoptosis [48]. Further study from unpublished data confirmed that kenaf seed oil causes cells death of human leukaemia HL60 and K562 cells and murine myelomonocytic WEHI-3H cell lines through induction of apoptosis [30].

### 4.2. Ginseng Root

It is one of the common herbal medications with multipharmaceutical functions in the United States and East Asia [49]. Ginsenosides are considered as the main active ingredients responsible for the pharmaceutical activities of ginseng root [50]. Ginsenosides are in a family of steroid saponins. Two major groups of ginsenosides contain protopanaxadiol and protopanaxatriol [51]. Several ginsenosides have been reported to exert anticancer effects ascribed to their ability to inhibit DNA synthesis, angiogenesis, and invasion, as well as induce cell cycle arrest and apoptosis [49]. For leukaemia, ginsenoside Rh1 showed a suppressive effect on the MAPK signaling pathway, resulting inhibition of invasion and migration of THP-1 acute monocytic cells [31].

### 4.3. *Euphorbia formosana* Hayata (EF)

It is a Taiwanese plant used to treat rheumatism, liver cirrhosis, herpes zoster, scabies, and photo aging, along with tumor suppression. However, the mechanisms by which it suppresses tumors have not been explored. Studies showed that a hot water extract of* Euphorbia formosana* (EFW) selectively inhibited the growth of human leukaemic cancer cells more than other solid human cancer cell lines. This inhibition was observed through the cell cycle phases where there was an increased in the S-phase, indicating cell dead, when THP-1 leukaemic cells were treated with 50–100 *µ*g/mL of EFW for 24 hours, while an increased in concentration (200–400 g/mL) led to the accumulation of the cells in the G0/G1 phase of the cycle whereas the plant extract had limited toxicity to healthy peripheral blood mononuclear cells (PBMCs). The effectiveness of EFW against THP-1 cells may be through caspase dependent apoptosis in leukaemic cells, which is mediated through the Fas and mitochondrial pathways. The potent antileukaemic activity of EFW* in vitro* warrants further investigation before treating leukaemia and other malignancies.

The ability of* Euphorbia formosana* (EF) to mediate proapoptotic activity intrigued to explore its possible applications as complementary and alternative medicine (CAM) for AML. Based on this evidence, researchers have found that EF induces apoptosis in many leukaemic cell lines [32].

### 4.4. Garlic (*Allium sativum*)

Garlic extract (GE) has a prominent role in the cancer prevention. It was well known because of its possible health benefits.* A. sativum* has free radical activity and its direct cytotoxic effect on cancer cells, particularly leukaemia [33]. The mechanisms by which garlic extract induces cytotoxic effects in cancer cells remain unknown. However, significant decreased of human leukaemia (HL-60) were observed when the cells were treated with GE in a concentration and time dependent manner. This finding demonstrates that at therapeutic concentrations, garlic treatment induced cytotoxic effects on HL-60 cells [52]* in vitro*.* Ajoene* as one of the noticed compounds in garlic has shown to inhibit proliferation and induce apoptosis of humans leukaemic cells and act as an antileukaemic agent for acute myeloid leukaemia therapy. The apoptosis activity of* ajoene* is via the mitochondria-dependent caspase cascade through a significant reduction of the antiapoptotic Bcl-2 that results in release of cytochrome C and the activation of caspase-3 [53].

Lamm and Riggs, 2001 [54] investigated the effect of garlic and two garlic compounds,* ajoene* and* allitridium*, compared with commonly used chemotherapeutic drugs on apoptosis of ALL cells and normal lymphocytes* in vitro* from newly diagnosed ALL patients. Other researchers [55] had demonstrated its functions as an antioxidant, by inhibiting the release of superoxide.

*Moringa oleifera (The Miracle Tree)* is a multipurpose plant, which has excessive use (preventative and treatment). The roots of this tree are used to treat malaria, hypertension, and stomach disorders, to expel a retained placenta and also it is a treatment for asthma and diabetes. Previous studies showed it had antileukaemic potency. Different form of* Moringa oleifera* extracts, hot water form, cold water, and ethanolic form, were prepared to test its antiproliferative effects on AML cell lines. Amongst these prepared extracts, ethanolic was found to be more effective in killing 51% of these cells than the rest of the forms. In conclusion roots of* Moringa oleifera* contain active components that were easily dissolved in ethanol; so it could be used as natural antileukaemic medicines [34].

*Vernonia amygdalina* is an African medicinal plant which is known to have two anticancer agents'* vernodalin* and* vernolide*. The roots are the principal material for herbal medicine and it has activity against leukaemic cells.* Vernonia amygdalina's* leaves inhibited proliferation of some cancer cell types, acute myeloid leukaemia (AML), acute lymphoblastic leukaemia (ALL) [35].

Cold water, hot water, and ethanol extracts of the* in vitro* cultured roots of this plant were tested for their antioxidant activity and efficacy against leukaemia cells. All of these extracts showed significant antioxidant activity and could kill the majority (50–75%) of abnormal cells among primary cells harvested from 3 patients with acute lymphoblastic leukaemia (ALL) and 3 with acute myeloid leukaemia (AML). DNA fragmentation patterns were detected within treated cells and inferred targeted cell death by apoptosis. The metabolites within the extracts may act as tumour inhibitors that promote apoptosis. Therefore,* in vitro* root culture can be an alternative to collection from the wild, cultivation in the field or to the chemical synthesis of anticancer agents. Furthermore, the plant extracts may be used to supplement or replace established drug treatments. Previous studies showed there were significant changes of lymphoblast cells when treated with the extracts after 24 hours incubation. Response to these extracts was not basically dose and time dependent. Also, to reaffirm and look for its effects on normal cells, a normal mononuclear cells obtained from healthy volunteer were tested and no significant effects were observed. Therefore, the extract has more effects on leukaemic cells than the normal [56].

### 4.5. *Achillea fragrantissima* (*Af*)

The genus* Achillea*, consisting of 140 perennial herbs, has traditionally been used in Middle Eastern countries.* A. fragrantissima* has analgesic, antiulcer, hepatoprotective, and wound healing activities [57].* Af* has also been shown to possess strong antioxidant potential [36].* Af* extract has the anticancer properties on CML cell line K562 (human chronic myelogenous leukaemia)* in vitro* model. Studies indicated that* Af* extract induced morphological changes involving spherical to the spindle and elongated shapes in K562 and Jurkat cells representing differentiation and cell cycle arrest, respectively. This extract canreduce the proliferation and causes cell death in K562 and also may serve as a novel potential therapeutic capable of inducing differentiation, cell cycle arrest, and apoptosis in chronic myelogenous leukaemia (CML) cells [58].

### 4.6. *Typhonium flagelliforme*

It is a multipurpose herbwhich belongs to the Araceae (Arum) family and is known as “rodent tuber” in Malaysia. This plant is cultured in the South East Asian countries, the southern part of India, Seri Lanka, and Australia. The plant has curative properties against a variety of illnesses, including injuries, edema, pulmonary ailments, and bleeding. Lai et al. [59] have reported that* T. flagelliforme* can act as anticancer and antiproliferative activity* in vitro*. The plant is also one of the best herbal remedies in Malaysia. Choo et al. [60] performed an experiment which proved cytotoxic activity on murine P388 leukaemia cell line. Cytotoxicity studies of* T. flagelliforme* were also performed* in vitro* against human T3-lymphoblastoid cell lines (CEM-ss) and significant effects were observed [37]. They also further investigated the effects of the leaves extract in* in vivo* study using BALB/c leukaemic mice model and found that there were significant reductions in the cell count of immature granulocytes and monocytes when the TF extract is orally administered for 28 days at different doses of 200, 400, and 800 mg/kg.

### 4.7. Grape Seeds

They are products from whole grape seeds which are rich in vitamin E, flavonoids, linoleic acid, and phenolic OPCs. Based on research from the University of Kentucky, grape seed extract causes commit cell suicide* in vitro*. They found that within 24 hours, 76 percent of leukaemic cells had died after being exposed to the extract. Grape seed extract activates JNK, a protein that regulates the apoptotic pathway, and it leads to cell death or apoptosis. Grape seed extract has shown activity in cancer cell lines, including skin, breast, colon, lung, stomach, and prostate cancers. Epidemiological evidence presented that eating vegetables and fruits helps to end cancer development.

Shi had exposed leukaemic cells to the extract in different doses and had noted it can cause apoptosis in these cells at one of the higher doses. They also discovered that the extract does not affect normal cells. They found that the extract strongly activated the JNK pathway, which then led to upregulation of Cip/p21, which controls the cell cycle. They checked this finding by using an agent that inhibited JNK and found that the extract was ineffective by silencing the JNK gene. They determined that grape seed extracts had a fatal attack in leukaemic cell line [46].

### 4.8. Pomegranate

It has been found as anticancer agents. Pomegranate juice (PGJ) induced apoptosis by altering in the cell cycle [39]. Treatment of four leukaemic cell lines with five fractions (fractions are A) unbound fraction, ultrapure water (fraction B), acetonitrile (fraction C), acetone (fraction D), and ethyl acetate (fraction E), respectively, obtained from PGJ by solid phase extraction, demonstrated that only the acetonitrile fractions decreased adenosine triphosphate (ATP) levels in all leukaemic cell lines [61]. Acetonitrile fractions also significantly activated caspase-3 and induced nuclear morphology characteristic of apoptosis. S-phase arrest was induced by acetonitrile fractions which matched S-phase arrest seen previously following whole PGJ treatments. The acetonitrile fractions contained higher phenol content than the whole PGJ whereas only low levels of phenols were seen in other fractions. Liquid chromatography mass spectrometry (LC-MS) analysis revealed that acetonitrile fractions were enriched in ellagitannins, ellagic acid, and hydroxycinnamic acid derivatives but depleted in anthocyanins. Individual treatments with identified compounds demonstrated that the ellagitannin, punicalagin, was the most active and mimicked the responses seen following acetonitrile fraction treatment. Bioactive components within pomegranate were confined to the acetonitrile fraction of PGJ. The enrichment in ellagitannins and hydroxycinnamic acids suggests these may provide the majority of the bioactivities of PGJ. Individual treatments with compounds, identified, demonstrated that the ellagitannin, punicalagin, was the most active agent, highlighting this compound as a key bioactive agent in PGJ [39].

Overwhelming evidence indicated that consumption of fruits and vegetables with antioxidant properties correlates with a reduced risk of cancers, including leukaemia.

### 4.9. Carrot

The carrot also has been found to have a good effect on leukaemia. Carrot contains beneficial agents, such as *β*-carotene and polyacetylenes, which could be effective in the treatment of leukaemia [40]. Leukaemia cell lines and nontumor control cells were treated with carrot juice extracts for 72 hours* in vitro*. The treatment of leukaemic cell lines with carrot juice had shown that extracts from carrots can induce apoptosis and cause cell cycle arrest in leukaemic cell lines. The findings suggest that carrots may be an excellent source of bioactive chemicals for the treatment of leukaemia [40].

*Ganoderma lucidum (G. lucidum)* is a medicinal mushroom having biological effects such as immune modulation and antitumour actions. In China and many other Asian countries,* G. lucidum* is used as a folk remedy to promote health and longevity. Many studies have shown that* G. lucidum* modulates the immune system, by antigen-presenting cells, natural killer (NK) cells, and the T and B lymphocytes. Chang et al. [41] studied the effect of* G. lucidum* on promoting immune responses in BALB/c mice injected with WEHI-3 leukaemic cells. They found that there were increases in the percentages of CD3 and CD19, but decreases in the percentages of Mac-3 and CD11 markers, suggesting that differentiation of the precursor of T and B cells was promoted but macrophages were inhibited.* G. lucidum* can decrease the weight of spleens as compared with control mice. It is also shown to promote phagocytosis by macrophage from peripheral blood mononuclear cell (PBMC) as well as natural killer cell activity and improvement of blood circulation. It also decreased the percentage of leukaemic cells in the spleens of mice before they were injected with WEHI-3 cells [41].

### 4.10. *Berberis vulgaris* (Barberry)

Barberry plants, including* Berberis aristata*,* Berberis aquifolium*,* Berberis asiatica*,* Berberis croatica*,* Berberis thunbergii*,* and Berberis vulgaris*, are shrubs mainly grown in Asia and Europe, especially in India and Iran. Their roots, barks, leaves, and fruits are often used as folk medicine [42, 43, 62]. The stem, root bark, and fruit of barberry contain isoquinoline alkaloids (e.g., berberine), which are the main active ingredients of barberry [63]. The amount of berberine fractions in the stem was 2 : 3 times higher than the amount in the leaves. It is a natural isoquinoline alkaloid with an intense yellow color and a bitter taste. It is found in many medicinal plants used in traditional Indian and Chinese medicine.

Researchers found that berberine is mainly distributed in the roots, barks, and stem of plants. For decades, berberine has intrigued increasing interest in its significant bioactivities, such as antioxidant, antimicrobial, and anticancer effects.

Berberine could suppress the growth and proliferation of different kinds of cancer cells. It can induce cell cycle arrest at different cell cycle phases, mainly at G0/G1 checkpoint via inhibiting the expression of cyclin D1 in different types of cancer cells by influencing of p53 [64–66] and regulates by increasing the expression of Cclk inhibitory proteins (Cdki), such as Cip1/p21 and Kip1/p27, inhibiting the expression of cyclin-dependent kinase (Cdk) 2, Cdk4, and Cdk6 and cyclins D1, D2, and E, as well as enhancing the binding of Cdki to Cdk [67, 68]. In addition, G1/S and G2/M phase cell cycle arrests were involved in berberine-induced cell cycle arrest. In HL-60 cells, berberine caused cell accumulation in S-phase via a strong activation of Chk2, phosphorylation and degradation of Cdc25A, and inhibition of Cdc2 (CDK1) and the proto-oncogene cyclin D1 [69]. Berberine also can inhibit tumor growth* in vivo* through inhabitation of the N-acetyltransferase, cyclooxygenase-2 (COX-2), and topoisomerases [70]. It also noted to have anticancer activity against various WEHI-3 leukaemic cells where it could induce apoptosis via activation of caspase-3 and inhibition of topoisomerases-II [71].

## 5. Conclusion

There is an ocean of knowledge about medicinal plants, but still only a few pearls have been searched as therapeutic agents. This review article elaborated different species of plants and fruits used as traditional medicines against leukaemia. Studies suggested that herbal medicines have a great potential in combating leukaemia. These herbs and fruits could be the best candidate for future leukaemia therapy with minimal adverse effects, easier availability, and better acceptability as compared to chemotherapy and probably they will provide more potent antileukaemic agents in future.

## Figures and Tables

**Table 1:** Major biological properties of herbs attributed to potential antileukaemic effect.

Herb names	Activity/uses	Human/animal subjects	Results	References
*Hibiscus cannabinus* (*Kenaf*)	Antioxidative Antileukaemic	WEHI-3B HL-60 K562	Induced apoptosis in WEHI-3B, HL-60, and K562 cells	Warner et al., 1969 [30] He and Na, 2001

*Ginseng root *	(i) Induce cell cycle (ii) Arrest apoptosis (iii) Effect on the MAPK signaling pathway	THP-1 acute monocytic cells	Inhibition of invasion and migration of THP-1 acute monocytic cells	Choi et al., 2011 [31] Yan et al., 2013

*Euphorbia formosana *	Antileukaemic	THP-1 and leukaemic cell lines	(i) To treat leukaemia and other malignancies (ii) Induces apoptosis in various leukaemic cell lines	Hsieh et al., 2013 [32] Mijatovic et al., 2011 [29]

*Allium sativum* (*garlic*)	Antileukaemic	Leukaemic cell lines	Direct cytotoxic effect on cancer cells With a free radical activity	Abdullah et al., 1988 [33]

*Moringa oleifera *	(i) Antileukaemic (ii) Antiproliferative	AML cell lines	Ethanolic extract killed most of the leukaemic cells	Eltayb et al., 2010 [34]

*Vernonia amygdalina *	Antiproliferative	AML & ALL cell lines	(i) Showing remarkable damage of lymphoblasts with plant extract in leukaemic cell (ii) Inhibiting proliferation of some leukaemic cell lines	El-Shemy et al., 2007 [35]

*Achillea fragrantissima *	Hepatoprotective antileukaemic	CML cell line (K562)	(i) Cell cycle arrest (ii) Apoptosis in CML cells	Tarawneh et al., 2010 [36]

*Typhonium flagelliforme *	(i) Therapeutic (ii) Antiproliferative (iii) Cytotoxic (iv) Antileukaemic	CEMss WEHI-3 P388	(i) Antileukaemic effect on mice model (ii) *In vitro* cytotoxic effect of leaves and tubers of *T. Flagelliforme* extract against human T4-lymphoblastoid cell line CEM-ss	Mohan et al., 2010 [37]

**Table 2:** Biological properties of some fruits with antileukaemic effect.

Names of fruits	Activity/uses	Human/animal subjects	Results	References
*Grape seed *	(i) Cell death (ii) Apoptosis	Leukaemic cells	(i) Forces leukaemia cells to commit cell suicide (ii) Activation of the JNK pathway	Ning et al., 2009 [38]

*Pomegranate *	(i) Antioxidant (ii) Antileukaemic	Leukaemia cell lines	The acetonitrile fractions decreased (ATP) levels in all leukaemic cell lines	Dahlawi et al., 2013 [39]

*Carrot *	Strong source of bioactive chemicals for the treatment of leukaemia	Leukaemia cell lines	Antileukaemic	Zaini et al., 2011 [40]

*Ganoderma lucidum *	Antitumour	WEHI-3 leukaemic cells (BALB/c mice)	(i) It decreased the weight of spleens as compared with control mice (ii) To increase the percentages of CD3 and CD19 but decrease the percentages of Mac-3 and CD11b markers	Chang et al., 2009 [41]

*Berberis vulgaris* (berberine)	(i) Antioxidant (ii) Antimicrobial (iii) Anticancer	HL-60 & WEHI-3	(i) Suppress the growth and proliferation of different HL-60 (ii) Induced G1-phase cell cycle arrest (iii) Influence p53 (iv) Anticancer activity against leukaemic cells (WEHI-3)	Andola et al., 2010 [42] Kulkarni and Dhir 2010 [43]
